# Successful endoscopic submucosal dissection of colorectal lipoma with an overlying adenoma

**DOI:** 10.1002/jgh3.12901

**Published:** 2023-05-26

**Authors:** Akito Furuta, Shunsuke Omoto, Taro Inoue, Mitsuru Yanai, Hideo Iwabe, Hiroshi Takihara, Kohei Ishibashi, Hironori Tanaka, Ko Matsuura, Shunsuke Ogata, Akitaka Yokomura, Masato Hoshikawa, Michihito Kono, Takasi Koriyama, Tomohiko Tazawa, Eri Tsuyuguchi, Yasuo Yamasaki, Shun Esumi, Yoshimasa Tsuruta, Takahiro Shishimoto, Masaki Yamamoto, Wataru Ono

**Affiliations:** ^1^ Department of Gastroenterology Kishiwada Tokushukai Hospital Kishiwada Japan; ^2^ Department of Gastroenterology Kindai University Faculty of Medicine Osaka‐sayama Japan; ^3^ Department of Pathology Sapporo Tokushukai Hospital Sapporo Japan; ^4^ Department of internal medicine Kamagaya General Hospital Kamagaya Japan; ^5^ Department of Gastroenterology Uji Tokushukai Hospital Uji Japan

**Keywords:** endoscopic submucosal dissection, lipoma, lipoma with an overlying adenoma

## Abstract

We report the case of a 65‐year‐old woman whose colonoscopy revealed a soft submucosal tumor approximately 7 cm in diameter in the ascending colon with an overlying flat lesion. The tumor was diagnosed as a lipoma with an overlying adenoma. Endoscopic submucosal dissection (ESD) was performed. Pathological examination revealed that the epithelium was a low‐grade tubulovillous adenoma, while the submucosal yellow tumor was a lipoma. ESD appears to be a safe and effective treatment for colorectal lipomas overlying lipomas with colorectal adenomas.

## Graphical abstract

We report a case of endoscopic submucosal dissection (ESD) of a 7 cm lipoma in the ascending colon with an overlying flat colorectal adenoma. ESD appears to be a safe and effective treatment for an overlying colorectal lipoma with colorectal adenoma.

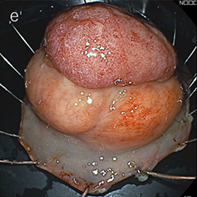

## Introduction

Colorectal lipoma is indicated for treatment of symptomatic cases and cases in which large size and malignancy cannot be ruled out. Surgery has been performed for colorectal lipomas, but in recent years, safer resection by less invasive endoscopic submucosal dissection (ESD) has been reported. Here we report a case in which a colorectal lipoma with an overlying adenoma was resected by ESD, which has not been reported so far.

## Case Report

**Figure 1:**
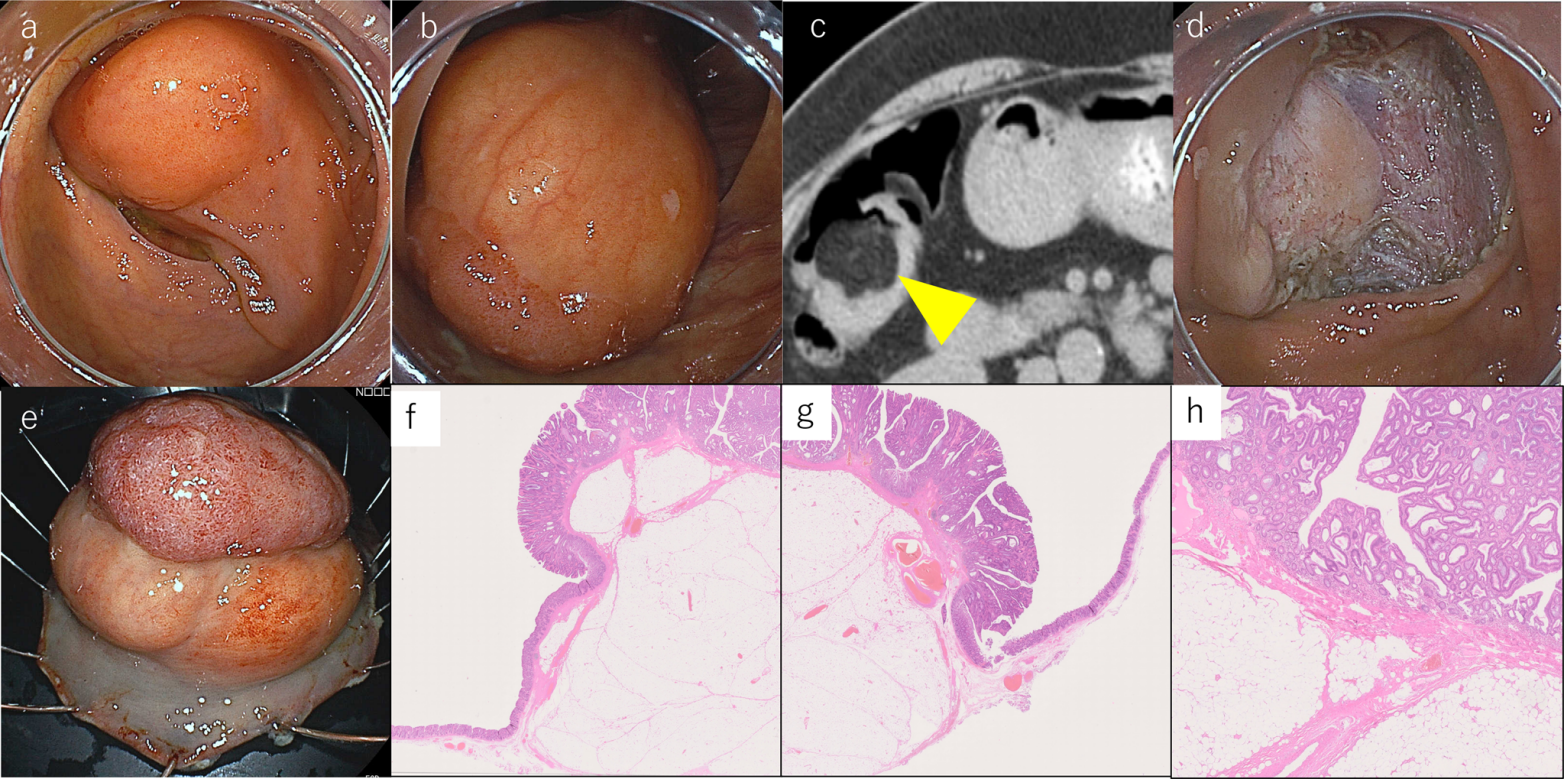
(a, b) Colonoscopy revealed a soft and yellowish submucosal tumor approximately 7 cm in diameter in the ascending colon, with a flat lesion overlying the tumor. (c) Computed tomography (CT) revealed a tumor in the ascending colon (yellow arrow), and the CT density within the tumor was the same as that of the fatty tissue. (d, e) Once endoscopic submucosal dissection (ESD) was indicated, ESD was performed and the tumor was resected en bloc. (f–h) The epithelium was a low‐grade tubulovillous adenoma, while the submucosal yellow tumor was a lipoma. The boundaries between the two are clearly demarcated (H&E stain).

A 65‐year‐old woman presented with a positive fecal occult blood test result. Colonoscopy revealed a soft and yellowish submucosal tumor approximately 7 cm in diameter in the ascending colon, with a flat lesion overlying the tumor (Fig. 1a,b). The flat lesion, classified as type 2A on narrow‐band imaging (NBI) based on the Japan NBI Expert Team (JNET) classification, led to a diagnosis of colorectal adenoma. Computed tomography (CT) revealed a tumor in the ascending colon, and the CT density within the tumor was the same as that of the fatty tissue (Fig. 1c). Based on these findings, the tumor was diagnosed as a lipoma with an adenoma. Once ESD was indicated, it was performed, and the tumor was resected en bloc (Fig. 1d,e) Further, no complications were observed either during or after the procedure.

Pathological examination was performed using a 75 × 55 mm specimen. The epithelium was a low‐grade tubulovillous adenoma, while the submucosal yellow tumor was a lipoma. The boundaries between the two were clearly demarcated (Fig. 1f–h).

## Discussion

Colorectal lipoma is indicated for treatment of symptomatic cases and cases in which large size and malignancy cannot be ruled out. In this case, the complication caused by the adenoma warranted treatment. Although surgery has been the conventional treatment for such lipomas, recent reports^1^ and single‐center studies^2^ have demonstrated safe resection by ESD. Since ESD is less invasive than surgery, we chose to perform ESD in this patient. ESD requires advanced endoscopic skills compared to other endoscopic treatments; however, it allows precise dissection of the submucosa between the muscle layer and the lipoma since the incision line is directly visible.^3^

Although ESD of colorectal lipomas has been reported, there are only a few reports of lipomas covering adenoma,^4^, ^5^ and there is lack of literature on ESD of lipomas with overlying adenoma. In our patient, we concluded that the adenoma was located on top of the lipoma based on CT and endoscopic images, which we then resected en bloc using ESD. Thus, ESD might be a safe and effective treatment for colorectal lipomas as well as lipomas with overlying colorectal adenomas.

## Patient consent statement

Informed consent was obtained from the patient for publication of this case report and accompanying images.
